# Association of genetic variations in the Wnt signaling pathway genes with myocardial infarction susceptibility in Chinese Han population

**DOI:** 10.18632/oncotarget.10401

**Published:** 2016-07-04

**Authors:** Jing Tao, Yong-tao Wang, Mayila Abudoukelimu, Yi-ning Yang, Xiao-mei Li, Xiang Xie, Bang-dang Chen, Fen Liu, Chun-hui He, Hua-yin Li, Yi-tong Ma

**Affiliations:** ^1^ Department of Cardiology, the First Affiliated Hospital of Xinjiang Medical University, Urumqi, Xinjiang, China; ^2^ Xinjiang Key Laboratory of Cardiovascular Disease Research, Urumqi, Xinjiang, China

**Keywords:** myocardial infarction, Wnt signaling pathway, polymorphism, susceptibility, Pathology Section

## Abstract

Numerous studies have implicated the Wnt pathway in the development and progression of myocardial infarction (MI); however, there are very few investigations addressing the effects of polymorphisms in the Wnt pathway genes on MI susceptibility. We investigated the possible correlation between genetic variations in Wnt pathway genes and MI risk. Three polymorphisms (rs7832767 C > T in *SFRP1* gene, rs2293303 C > T in *CTNNB1* gene, rs16893344 C > T in *WISP1* gene) were finally selected and genotyped in 465 MI patients and 485 healthy controls, using the PCR-RFLP method. We found that the *SFRP1* rs7832767 variant allele (T) was associated with a significantly increased risk of MI [TT vs. CC: adjusted odds ratio (AOR) = 3.13, 95% CI = 1.78-5.51; CT/TT vs. CC: AOR = 1.53, 95% CI = 1.12-2.08; TT vs. CC/CT: AOR = 2.87, 95% CI = 1.66-4.97)]. The significant association with MI risk was also found for the *CTNNB1* rs2293303 (CT vs. CC: AOR = 3.48, 95% CI = 2.28-5.33; TT vs. CC: AOR = 7.37, 95% CI = 2.08-26.16; CT/TT vs. CC: AOR = 3.72, 95% CI = 2.46-5.62; TT vs. CC/CT: AOR = 5.52, 95% CI = 1.58-19.28), and *WISP1* rs16893344 polymorphisms (CT vs. CC: AOR = 2.43, 95% CI = 1.70-3.47; TT vs. CC: AOR = 5.17, 95% CI = 1.85-14.41; CT/TT vs. CC: AOR = 2.58, 95% CI = 1.83-3.66; TT vs. CC/CT: AOR = 3.88, 95% CI = 1.41-10.64). The associations remain significant in stratified analysis by demographic and clinical characteristics of participants, with few exceptions. Our study provided the first evidence of the association between polymorphisms in the Wnt pathway genes and MI susceptibility in Chinese Han population. Epidemiological studies with larger samples and functional analyses are warranted to further verify our results.

## INTRODUCTION

Myocardial infarction (MI), frequently resulting from coronary thrombosis, has been the major cause of death in humans worldwide, and its incidence is still rapidly increasing in low-, moderate-, and high-income countries [1, 2], Finland and Japan have the highest and lowest MI incidence rate worldwide, respectively [3-5]. The World Bank estimates that by 2030, 23 million Chinese patients will experience accurate MI annually [6]. Therefore, this disease is a serious challenge facing the nation's healthcare system. There are some conventional predisposing factors for the development of MI, involving tobacco use, diabetic diseases, abnormally elevated blood pressure, hypercholesterolemia, and obesity [7]. Aside from these modifiable risk factors, accumulating studies have demonstrated that MI is a complicated polygenic disease, arising from some different causes and impacts, which is believed to be a result of interactions between an individual's genetic composition and a variety of environmental risk factors [8-10]. Moremore, some patients with MI might have never exposed to any well-established risk factors. On the contrary, only a fraction of individual exposed to risk factors eventually develop this type of disease in the lifetime. Therefore, genetic factors, such as some uncharacterized genetic component may predispose to MI [11]. Abundant studies have identified some molecular pathways that are associated with MI, including Notch [12], MAPK [13], as well as NF-kB [14] signal transduction pathways. Some genetic alterations (e.g., mutation, gene amplification, and genetic variation) within signaling pathways may contribute to MI susceptibility and also serve as druggable targets for the disease. The identification of pathogenic genetic alterations may lead to new understandings of the development of MI.

In the canonical Wnt signaling pathway, Wnt ligands bind to receptors (e.g., the seven-pass transmembrane Frizzled receptor) and lead to the stabilization of intracellular β-catenin, which permits accumulation of β-catenin and its nuclear translocation, subsequently influencing the association of transcription factors (e.g., Tcf/LEF) with transcriptional co-repressors to activate Wnt-dependent target gene expression, such as Wnt-1 inducible signaling protein 1 (WISP1) [15-17]. On the other hand, the secreted frizzled-related protein (SFRP) family (SFRP1-5) can competitively bind to Wnt proteins and block their interactions with Frizzleds, negatively regulating the activation of canonical Wnt signaling pathway and expression of downstream genes [18, 19]. Compelling evidences have indicated that the Wnt signaling pathway is involved in MI [20, 21], but the underlying mechanisms remain to be further clarified.

Thus far, there are few epidemiologic studies evaluating the association between genetic variations in the Wnt pathway genes and the risk of MI. To precisely determine the association of the Wnt pathway gene polymorphisms with MI risk, we investigated the genotype distributions of several single nucleotide polymorphisms (SNPs) in key genes within this important pathway (i.e., rs7832767 C > T in *SFRP1* gene, rs2293303 C > T in *CTNNB1* gene, rs16893344 C > T in *WISP1* gene) in the case-control study with 465 MI cases and 485 controls.

## RESULTS

### Characteristics of study participants

Demographic and clinical characteristics of all participants were listed in Table 1. In total, 465 patients with MI and 485 healthy controls were enrolled in this study. Of the MI patients, 255 (54.84%) were women and 210 (45.16%) were men, with mean age 67.32±8.57 years (range, 37-89 years). As to the controls, 273 (56.29%) were women and 212 (43.71%) were men, with mean age 66.98±9.07 years (range, 33-91 years). We did not observe significant differences between patients and controls regarding age (*P* = 0.552), sex (*P* = 0.653), drinking status (*P* = 0.742), body mass index (BMI) (*P* = 0.112), triglycerides (TG) (*P* = 0.618), high-density lipoprotein cholesterol (HDL-C) (*P* = 0.719), uric acid (*P* = 0.185), blood urea nitrogen (BUN) (*P* = 0.996), and creatinine (Cr) (*P* = 0.779). The patients and controls differed significantly with regards to systolic blood pressure (SBP) (*P* < 0.0001), diastolic blood pressure (DBP) (*P* < 0.0001), fasting plasma glucose (FPG) (*P* < 0.0001), total cholesterol (TC) (*P* < 0.0001), low-density lipoprotein cholesterol (LDL-C) (*P* < 0.0001), the prevalence of hypertension (*P* < 0.0001), diabetes (*P* < 0.0001), dyslipidemia (*P* = 0.017), smoking (*P* = 0.0002), and obesity (*P* = 0.028). In terms of these clinical parameters, cases tended to be unhealthier than controls.

**Table 1:** Demographic and clinical characteristics of study participants

Variables	Case (*n* = 465)		Control (*n* = 485)		*P*^a^
	No	%	No	%	
Age range, yr	37-89		33-91		0.552
Mean ± SD	67.32 ± 8.57		66.98 ± 9.07		
≤ 60	88	18.92	92	18.97	
61-70	191	41.08	192	39.59	
71-80	161	34.62	182	37.53	
>80	25	5.38	19	3.92	
Sex					0.653
Female	255	54.84	273	56.29	
Male	210	45.16	212	43.71	
Smoking status					0.0002
Never	308	66.24	374	77.11	
Ever	157	33.76	111	22.89	
Drinking status					0.742
Never	414	89.03	435	89.69	
Ever	51	10.97	50	10.31	
BMI (kg/m2)	26.07 ± 4.06		25.66 ± 3.83		0.112
<18.5	9	1.94	12	2.47	
18.5-23.9	147	31.61	151	31.13	
24.0-29.9	230	49.46	264	54.43	
≥30.0	79	16.99	58	11.96	
Hypertension					<0.0001
No	312	67.10	231	47.63	
Yes	153	32.90	254	52.37	
Diabetes					<0.0001
No	263	56.56	45	9.28	
Yes	202	43.44	440	90.72	
Dyslipidemia					0.017
No	198	42.58	244	50.31	
Yes	267	57.42	241	49.69	
Obesity					0.028
No	79	16.99	58	11.96	
Yes	386	83.01	427	88.04	
SBP (mmHg)	147.04 ± 23.48		137.36 ± 19.04		<0.0001
DBP (mmHg)	90.40 ± 19.34		84.86 ± 16.11		<0.0001
FPG (mmol/L)	6.74 ± 1.74		5.36 ± 1.76		<0.0001
TG (mmol/L)	1.55 ± 1.14		1.59 ± 1.28		0.618
TC (mmol/L)	4.79 ± 1.13		4.41 ± 1.21		<0.0001
HDL-C (mmol/L)	1.24 ± 0.45		1.23 ± 0.50		0.719
LDL-C (mmol/L)	2.95 ± 0.99		2.67 ± 0.84		<0.0001
Uric acid (umol/L)	300.93 ± 93.12		293.41 ± 80.81		0.185
BUN (mmol/L)	5.35 ± 1.62		5.34 ± 1.75		0.996
Cr (mmol/L)	76.64 ± 23.06		76.06 ± 38.91		0.779

BMI, body mass index; SBP, systolic blood pressure; DBP, diastolic blood pressure; FPG, fasting plasma glucose; TG, triglyceride; TC, total cholesterol; HDL-C, high density lipoprotein cholesterol; LDL-C, low density lipoprotein cholesterol; BUN, blood urea nitrogen; Cr, creatinine

a The *P* value of the continuous variables was calculated by the independent-sample t-test. The *P* value of the categorical variables was calculated by χ2 test

### The genotypes distribution of selected polymorphisms in MI cases and controls

The genotypes distribution of the three selected polymorphisms in patients with MI and healthy controls were indicated in Table 2. The genotype freqency distributions of the three SNPs in controls were in accordance with the Hardy-Weinberg equilibrium (*P* = 0.769 for rs7832767, *P* = 0.754 for rs2293303, and *P* = 0.743 for rs16893344).

**Table 2:** The distribution of genotypes and alleles of Wnt signaling pathway gene between myocardial infarction patients and controls

Genotype	Case (*n*, %)	Control (*n*, %)	*P* ^a^	Crude OR (95% CI)	*P*	Adjusted OR (95% CI) b	*P* ^b^
SFRP1 rs7832767 C>T							
CC	231 (49.68)	286 (58.97)		1.00		1.00	
CT	182 (39.14)	170 (35.05)		1.33 (1.01-1.74)	0.042	1.27 (0.91-1.76)	0.167
TT	52 (11.18)	29 (5.98)		2.22 (1.37-3.61)	0.001	3.13 (1.78-5.51)	<0.0001
Additive			0.002	1.42 (1.16-1.73)	0.0006	1.55 (1.23-1.97)	0.0002
Dominant	234 (50.32)	199 (41.03)	0.004	1.46 (1.13-1.88)	0.004	1.53 (1.12-2.08)	0.007
Recessive	413 (88.82)	456 (94.02)	0.004	1.98 (1.23-3.18)	0.005	2.87 (1.66-4.97)	0.0002
*CTNNB1* rs2293303 C>T							
CC	358 (76.99)	407 (83.92)		1.00		1.00	
CT	95 (20.43)	74 (15.26)		1.46 (1.04-2.04)	0.027	3.48 (2.28-5.33)	<0.0001
TT	12 (2.58)	4 (0.82)		3.41 (1.09-10.67)	0.035	7.37 (2.08-26.16)	0.002
Additive			0.009	1.55 (1.16-2.08)	0.003	3.25 (2.24-4.71)	<0.0001
Dominant	107 (23.01)	78 (16.08)	0.007	1.56 (1.13-2.16)	0.007	3.72 (2.46-5.62)	<0.0001
Recessive	453 (97.42)	481 (99.18)	0.036	3.19 (1.02-9.95)	0.046	5.52 (1.58-19.28)	0.007
*WISP1* rs16893344 C>T							
CC	314 (67.53)	369 (76.08)		1.00		1.00	
CT	136 (29.25)	109 (22.47)		1.47 (1.09-1.97)	0.011	2.43 (1.70-3.47)	<0.0001
TT	15 (3.23)	7 (1.44)		2.52 (1.01-6.25)	0.047	5.17 (1.85-14.41)	0.002
Additive			0.007	1.50 (1.16-1.93)	0.002	2.38 (1.75-3.25)	<0.0001
Dominant	151 (32.47)	116 (23.92)	0.003	1.53 (1.15-2.03)	0.004	2.58 (1.83-3.66)	<0.0001
Recessive	450 (96.77)	478 (98.56)	0.068	2.28 (0.92-5.63)	0.075	3.88 (1.41-10.64)	0.009

a *c*2 test for genotype distributions between myocardial infarction patients and controls

b Adjusted for age, gender, smoking, drinking status, BMI, SBP, DBP, FPG, TG, TC, Uric acid, HDL-C, LDL-C, BUN, Cr

Overall, we found that, compared to non-carriers, carriers of *SFRP1* rs7832767 T allele had a significantly elevated MI risk [TT *vs*. CC: adjusted odds ratio (AOR) = 3.13, 95% confidence interval (CI) = 1.78-5.51; CT/TT *vs*. CC: AOR = 1.53, 95% CI = 1.12-2.08; TT *vs*. CC/CT: AOR = 2.87, 95% CI = 1.66-4.97] after adjustment for age, gender, smoking, drinking status, BMI, SBP, DBP, FPG, TG, TC, Uric acid, HDL-C, LDL-C, BUN, and Cr. We also found that the significant association between *CTNNB* rs2293303 C > T polymorphism and an increased MI risk (CT *vs*. CC: AOR = 3.48, 95% CI = 2.28-5.33; TT *vs*. CC: AOR = 7.37, 95% CI = 2.08-26.16; CT/TT *vs*. CC: AOR = 3.72, 95% CI = 2.46-5.62; TT *vs*. CC/CT: AOR = 5.52, 95% CI = 1.58-19.28). Similarly, *WISP* rs16893344 C > T polymorphism was also shown to significantly increase the risk of MI (CT *vs*. CC: AOR = 2.43, 95% CI = 1.70-3.47; TT *vs*. CC: AOR = 5.17, 95% CI = 1.85-14.41; CT/TT *vs*. CC: AOR = 2.58, 95% CI = 1.83-3.66; TT *vs*. CC/CT: AOR = 3.88, 95% CI = 1.41-10.64).

### Stratified analysis

We performed stratification analyses in terms of age, sex, smoking and drinking status, hypertension, diabetic diseases, dyslipidemia, and obesity to evaluate how these variables modified the association between the SNPs (*SFRP1* rs7832767 C > T, *CTNNB* rs2293303 C > T and *WISP1* rs16893344 C > T) and MI risk (Table 3). The *SFRP1* rs7832767 CT/TT genotypes were shown to significantly increase MI risk in subjects of age≤67 and under (AOR = 2.31, 95% CI = 1.43-3.74), men (AOR = 1.98, 95% CI = 1.21-3.24), smokers (AOR = 3.05, 95% CI = 1.59-5.84), drinkers (AOR = 9.75, 95%CI = 2.64-36.10), subgroup with hypertension (AOR = 5.99, 95% = 3.10-11.56), subgroup with diabetes (AOR = 4.77, 95% CI = 3.09-7.35) and without diabetes (AOR = 0.32, 95% = 0.14-0.73), subgroup with dyslipidemia (AOR = 1.59, 95% CI = 1.04-2.41) as well as obesity (AOR = 1.50, 95% CI = 1.07-2.10). As to the *CTNNB* rs2293303 C > T polymorphism, we found that the increased risk remained statistically significant in all subgroups except for non-diabetes and non-obesity subgroups. Finally, we found that the association between the *WISP1* rs16893344 CT/TT genotype and MI risk was prominent in subjects at age of older than 67 (AOR = 3.58, 95% CI = 2.21-5.81), women (AOR = 2.87, 95% CI = 1.83-4.51), men (AOR = 1.97, 95% CI = 1.12-3.47), non-smokers (AOR = 1.96, 95% CI = 1.32-2.91), smokers (AOR = 6.99, 95% CI = 3.04-16.08), non-drinkers (AOR = 2.29, 95%CI = 1.60-3.29), drinkers (AOR = 13.00, 95%CI = 2.44-69.25), non-hypertension (AOR = 1.88, 95% = 1.22-2.90), hypertension (AOR = 4.58, 95% = 2.38-8.82), diabetes (AOR = 9.83, 95% CI = 6.23-15.51), dyslipidemia (AOR = 2.73, 95% CI = 1.71-4.36) as well as obesity subgroups (adjusted OR = 2.80, 95% CI = 1.91-4.12).

**Table 3:** Logistic regression analysis for association of SNPs with MI risk in Wnt signaling pathway

Variables	rs7832767 (Case/Control)		Adjusted ORa	*P*^a^	rs2293303 (Case/Control)		Adjusted ORa	*P*^a^	**rs16893344 (Case/Control)**		Adjusted ORa	*P*^a^
	CC	CT/TT	95% CI		CC	CT/TT	95% CI		CC	CT/TT	95% CI	
Median age, yr												
≤67	96/133	112/72	2.31 (1.43-3.74)	0.0007	159/164	49/41	2.39 (1.30-4.37)	0.005	142/148	66/57	1.65 (0.98-2.77)	0.061
>67	135/153	122/127	1.15 (0.75-1.75)	0.529	199/243	58/37	5.36 (2.98-9.62)	<0.0001	172/221	85/59	3.58 (2.21-5.81)	<0.0001
Gender												
Females	127/155	128/118	1.20 (0.80-1.82)	0.379	183/232	72/41	4.36 (2.58-7.39)	<0.0001	162/211	93/62	2.87 (1.83-4.51)	<0.0001
Males	104/131	106/81	1.98 (1.21-3.24)	0.007	175/175	35/37	2.88 (1.43-5.80)	0.003	152/158	58/54	1.97 (1.12-3.47)	0.018
Smoking status												
Never	159/212	149/162	1.18 (0.82-1.70)	0.371	230/306	78/68	3.46 (2.18-5.50)	<0.0001	208/274	100/100	1.96 (1.32-2.91)	0.0008
Ever	72/74	85/37	3.05 (1.59-5.84)	0.0008	128/101	29/10	5.13 (1.92-13.75)	0.001	106/95	51/16	6.99 (3.04-16.08)	<0.0001
Drinking status												
Never	208/245	206/190	1.25 (0.90-1.74)	0.181	316/360	98/75	3.55 (2.31-5.44)	<0.0001	279/323	135/112	2.29 (1.60-3.29)	<0.0001
Ever	23/41	28/9	9.75 (2.64-36.10)	0.0006	42/47	9/3	11.77 (1.77-78.13)	0.011	35/46	16/4	13.00 (2.44-69.25)	0.003
Hypertension												
No	144/96	168/135	0.72 (0.49-1.06)	0.103	250/217	62/14	6.85 (3.49-13.47)	<0.0001	216/179	96/52	1.88 (1.22-2.90)	0.004
Yes	87/190	66/64	5.99 (3.10-11.56)	<0.0001	108/190	45/64	2.64 (1.39-5.00)	0.003	98/190	55/64	4.58 (2.38-8.82)	<0.0001
Diabetes												
No	185/21	78/24	0.32 (0.14-0.73)	0.007	244/41	19/4	1.21 (0.33-4.49)	0.777	237/29	26/16	0.21 (0.08-0.52)	0.0007
Yes	46/265	156/175	4.77 (3.09-7.35)	<0.0001	114/366	88/74	9.86 (5.91-16.46)	<0.0001	77/340	125/100	9.83 (6.23-15.51)	<0.0001
Dyslipidemia												
No	97/145	101/99	1.49 (0.93-2.40)	0.097	152/199	46/45	3.67 (1.96-6.89)	<0.0001	137/183	61/61	2.45 (1.42-4.23)	0.001
Yes	134/141	133/100	1.59 (1.04-2.41)	0.032	206/208	61/33	3.81 (2.15-6.74)	<0.0001	177/186	90/55	2.73 (1.71-4.36)	<0.0001
Obesity												
No	31/30	48/28	1.70 (0.73-3.95)	0.221	61/47	18/11	2.25 (0.78-6.53)	0.135	54/42	25/16	1.47 (0.58-3.72)	0.416
Yes	200/256	186/171	1.50 (1.07-2.10)	0.020	297/360	89/67	4.00 (2.54-6.32)	<0.0001	260/327	126/100	2.80 (1.91-4.12)	<0.0001

a Adjusted for age, gender, smoking, drinking status, BMI, SBP, DBP, FPG, TG, TC, Uric acid, HDL-C, LDL-C, BUN, Cr

## DISCUSSION

Identification of genetic variations that are able to modify individuals' susceptibility may advance the understanding of the pathophysiological characteristics of MI, and may further facilitate individualized treatment decision making [22]. In this case-control study, we genotyped SNPs in three major Wnt signaling pathway genes (*SFRP1, CTNNB1* and *WISP1*) in Chinese population, and investigated the correlation between these SNPs and MI risk. The results indicated that *SFRP1* rs7832767 C > T, *CTNNB1* rs2293303 C > T, and *WISP1* rs16893344 C > T were all strongly correlated with MI susceptibility.

The Wnt signaling pathway can be competitively inhibited by SFRP1 by sequestrating the Wnt ligands and consequently preventing the activation of downstream signaling cascade. Evidence from transgenic mice overexpressing SFRP1 revealed that overexpression of SFRP1 reduced infarct size and cardiac rupture in a coronary arter lighation-induced MI model and improves cardiac function, when compared to wild-type mice [23]. Previous investigations have revealed the associations of genetic variants in the *SFRP1* gene with tuberculosis, inflammation, and cancer susceptibility [24-26]. We explored the rs78332767 C > T polymorphism in the *SFRP1*gene and found that it was significantly associated with the risk of MI in Chinese Han population. Carriers of *SFRP1* rs78332767 TT genotype or CT genotype were at significantly increased risk of MI, suggesting *SFRP1* may be a susceptibility gene for the development of MI. To the best of our knowledge, this is the first study to scrutinize of the risk effect of *SFRP1* rs7832767 C > T in cardiovascular disease. Larger, well-designed studies, involoving different ethnical populations are warranted to validate our finding, and further functional analysis are also indispensable to provide biological supports for the causal association.

β-catenin encoded by the *CTNNB1* gene has multiple functions in the maintenance of cardiac tissue homeostasis. Stabilization of β-catenin has been related to adult cardiac hypertrophy, and down-regulation of this protein initiates heart formation in embryogenesis [27]. Aberrant action and excessively high expression levels of β-catenin have synergistic effects in various diseases. A number of animal experiments confirmed that β-catenin levels were significantly increased in the MI model [28]. There are several lines of evidence showing the contribution of *CTNNB1* rs2293303 C > T polymorphism to the risk and prognosis of cancer, elucitating its potential involvment the pathogenesis of human diseases. Wang et al. [29] demonstrated that carriers of polymorphic variant of *CTNNB1* rs2293303 C > T polymorphism had significantly increased risk of gastric cancer and survival advantage versus non-carriers in a Chinese population. Jia et al. reported the significant association between *CTNNB1* rs2293303 C > T and breast cancer susceptibility [30]. Our results indicated that *CTNNB1* rs2293303 C > T was correlated with MI, and carriers of variant T allele had a significantly increased MI risk. Our results suggested that this polymorphism might modify the function of β-catenin, thereby affecting the development and progression of MI. However, the exact underlying mechanism by which this polymorphism conferred MI susceptibility remains to be elucidated.

*WISP1* is a gene in the canonical Wnt signaling pathway [31]. Previous studies indicated that mutations in this gene were related to several diseases, involving lung cancer, asthma, hypertension, and spinal osteoarthritis [32-35]. It has been reported recently that genetic variations in the *WISP1* gene, including rs16893344 C > T, may confer lung cancer susceptibility [32]. We observed an association of *WISP1* rs16893344 C > T polymorphism with MI in Chinese Han population, with TT and CT risk genotype associated with an increase in MI risk. Overall, our findings may help to improve the understanding of the implication of inherited genetic factors in the onset and progression of MI.

Despite the promising findings in this study, several limitations should be mentioned. First of all, the sample size of this study is relatively moderate. The associations of MI risk with *SFRP1* rs7832767 C > T, *CTNNB1* rs2293303 C > T and *WISP1* rs16893344 C > T with MI should be confirmed studies with larger sample size, involving diverse ethnical group. Second, we only drawed conclusions based on a observational association study, and found that in MI patients, the major Wnt signaling pathway genes all had C > T nucleotide polymorphism. These was similar to the previous research about the prevalent C > T nucleotide polymorphism in various human cancers [36, 37], but the researcher further revealed that the C > T nucleotide alteration tends to change an arginine to another amino acid [37]. Thus, mechanistic studies should be performed to explore the functions of the three SNPs in the future. Third, we only investigated three SNPs. Another variants that may also modify MI susceptibility should be also considered, including SNPs positioned in the functional domain. To better understand the impact of genetic factors on the MI susceptibility, genetic analysis on other genes within Wnt pathway should be carried out.

Thus far, this study is the first attempt to explore the relation of MI susceptibility with genetic variants in the Wnt signaling pathway in Chinese Han population. Overall, the studied SNPs (*SFRP1* rs7832767 C > T, *CTNNB1* rs2293303 C > T, and *WISP1* rs16893344 C > T polymorphisms) were shown to significantly increase MI risk. These susceptibility loci in the major Wnt signaling pathway genes can be applied to prediction of individual genetic risk to MI, identify the high-risk subpopulation, and encourage prevention. In the future, large-scale case-control studies and functional analyses are essential to verify these findings.

## MATERIALS AND METHODS

### Ethical approval of the study protocol

This study was approved by the Ethics Committee of the First Affiliated Hospital in the Xinjiang Medical University, Xinjiang, China. We conducted the study according to the standards of the Declaration of Helsinki. All of the patients provided written informed consents and explicitly provided permission for DNA analyses, as well as the collection of relevant clinical data.

### Subjects

All patients with MI and control subjects were recruited from the First Affiliated Hospital of Xinjiang Medical University from 2007 to 2014. We enrolled a total of 465 patients who suffered from first MI. Moreover, 485 sex-and age-matched healthy individual served as control subjects. MI diagnosis was established by the presence of chest pain lasting 20 min combined with ST-segment elevation or pathological Q waves on a surface electrocardiogram [38]. Patients with MI had to exhibit either an angiographically occluded infarct-related artery or regional wall motion abnormalities corresponding to the electrocardiographic infarct localization or both. Patients were excluded if they presented with ascertained congenital hypercoagulable status, a proven disease limiting life expectancy, or declared cocaine abuse. The control subjects were selected from volunteers who had visited our hospital between 2007 and 2014 for regular medical check-ups and were found to be healthy. Individuals would be considered eligible disease-free controls if they had angiographically normal coronary arteries and if they had no history of MI, no symptoms suggestive of MI, no electrocardiographic signs of MI, no regional wall motion abnormalities, and no relevant valvular abnormalities in echocardiograms [39]. Coronary angiography in the control individuals was performed for the evaluation of chest pain. Control subjects with coronary heart disease and any neoplasm, cardiomyopathy or severe illness limiting life expectancy or refusing consent were excluded. The response rates of participants were 100%.

### Laboratory examination and definition of cardiovascular risk factors

Serum concentrations of TC, TG, FPG, HDL-C, LDL-C, BUN, Cr and uric acid were measured using standard methods in the Department of Clinical Laboratory of First Affiliated Hospital, Xinjiang Medical University as described previously [40, 41]. Major coronary artery disease (CAD) risk factors were defined based on current national guidelines. Dyslipidemia was defined as TG ≥ 2.26mmol/L, TC ≥ 6.22 mmol/L, LDL -C ≥ 4.14 mmol/L, HDL-C < 1.04 mmol/L, or a prior Dyslipidemia diagnosis and/or receiving a lipid-lowering drug [42]. Hypertension was defined as a SBP ≥ 140 mmHg, DBP ≥ 90 mmHg, or a prior hypertension diagnosis and/or receiving an antihypertensive drug [43]. Obesity was defined as a BMI ≥ 30.0 [44]. Diabetes was defined as FPG ≥ 6.99mmol/L, or a prior diabetes diagnosis and/or using a diabetes drug [45]. Smoking was defined as currently smoking cigarettes.

### DNA extraction

Blood samples were collected from all participants using a standard venipuncture technique and EDTA-containing tubes. DNA was extracted from the peripheral blood leukocytes using a whole blood genome extraction kit (Beijing Bioteke Corporation, Beijing, China) as described previously [40, 41].

### SNP selection

Candidate genes must be well characterized and key components of the Wnt signaling pathway in terms of functional significance. After carefully literature reviewing [26, 29, 32], we finally included three key genes (*SFRP1, CTNNB1* and *WISP1*) within Wnt signaling pathway. Using Haploview 4.2 software and International HapMap Project website phase I &II database (http://www.hapmap.org), we obtained one tagging SNP for each candidate gene, rs7832767 C > T in *SFRP1*, rs2293303 C > T in *CTNNB1*, and rs16893344 C > T in *WISP1*. All these SNPs have a minor allele frequency (MAF) of ≥ 0.05 and not in linkage disequilibrium with each other (*r*^2^ < 0.8).

### Genotyping

**Figure 1:**
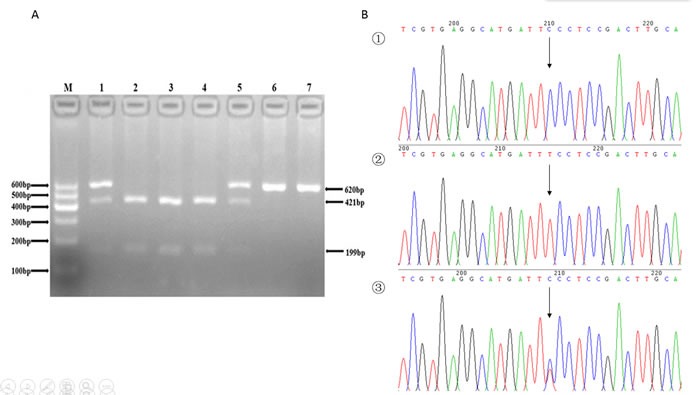
**A.** The restriction fragment length polymorphism analysis to determine rs7832767 C > T polymorphism, the CC genotype shows three bands at 56, 199, 421 bp (2, 3 and 4); the TT genotype shows two bands at 56 bp and 620 bp (6 and 7); and the CT genotype shows four bands at 56, 199, 421 and 620 bp (1 and 5). **B.** Nucleotide sequences around rs7832767 C > T polymorphism ((1), CC genotype; (2), TT genotype; (3), CT genotype).

**Figure 2:**
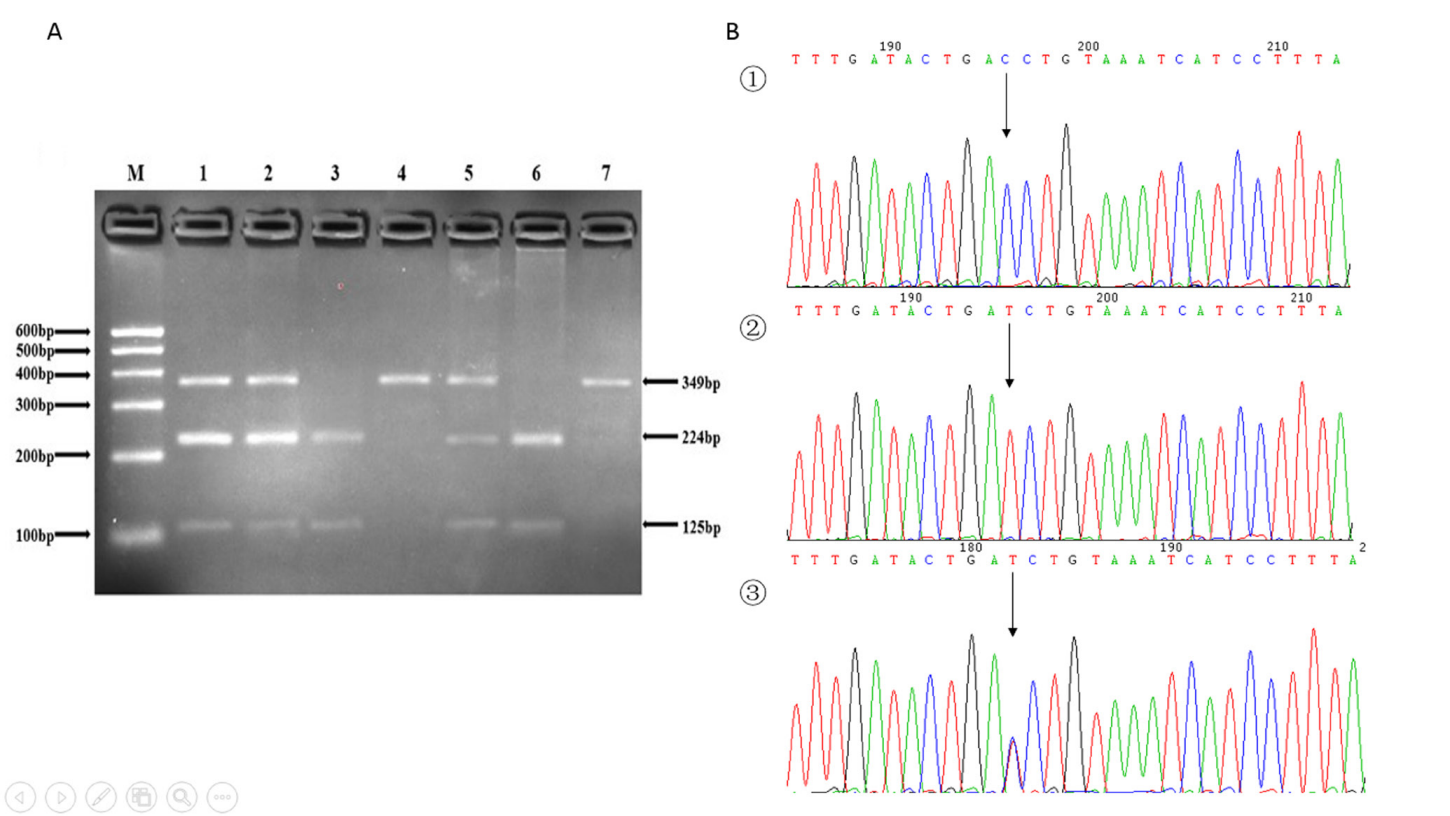
**A.** The restriction fragment length polymorphism analysis to determine rs2293303 C > T polymorphism, the CC genotype shows one 349 bp band (4 and 7); the TT genotype shows two bands at 125 bp and 224 bp (3 and 6); and the CT genotype shows three bands at 125 bp, 224 bp and 349 bp (1, 2 and 5). **B.** Nucleotide sequences around rs2293303 C > T polymorphism ((1), CC genotype; (2), TT genotype; (3), CT genotype).

**Figure 3:**
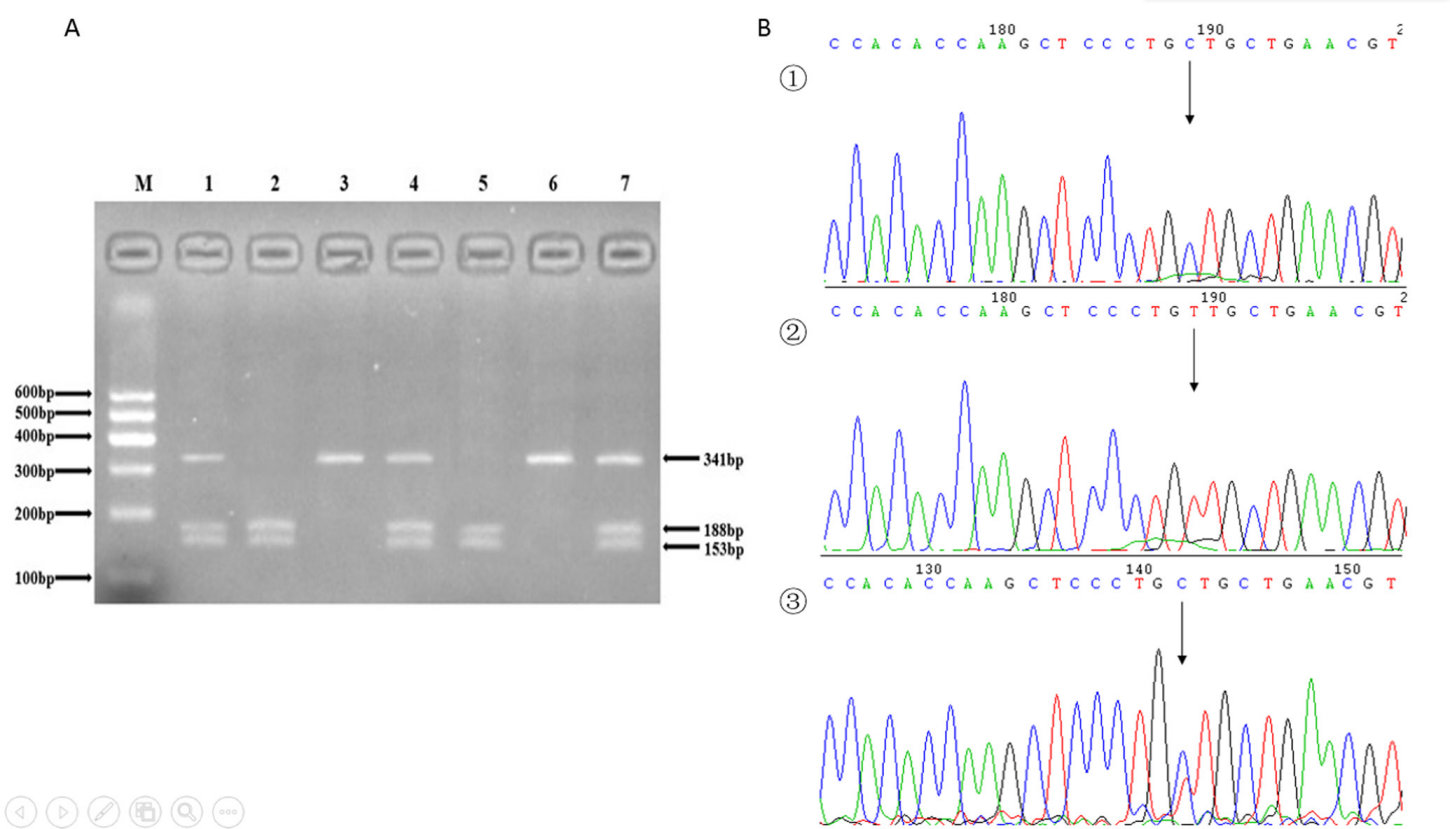
**A.** The restriction fragment length polymorphism analysis to determine rs16893344 C > T polymorphism, the CC genotype shows two bands at 153 bp and 188 bp (2 and 5); the TT genotype shows one 341 bp band (3 and 6); and the CT genotype shows three bands at 153 bp, 188 bp and 341 bp (1, 4 and 7). **B.** Nucleotide sequences around rs16893344 C > T polymorphism ((1), CC genotype; (2), TT genotype; (3), CT genotype).

The genotyping in the present study was performed via polymerase chain reaction-restriction fragment length polymorphism (PCR-RFLP) analysis. We designed the sequencing primers using Primer Premier 5.0. The primers were synthesize by Shanghai Generay Biological Technology Company Limited (Shanghai, China). The PCR amplification was performed in a 50 μL final reaction volume, containing 25μL of 2*powder Taq PCR master mix (Beijing Biotech, Beijing, China), 50 ng of genomic DNA, 21 μL of distilled water, and 1 μL of each forward and reverse primer. The thermal cycling conditions were as follows: (1) *SFRP1*: an initial denaturation step at 95°C for 5 min, 35 cycles of 95°C for 30 s, 51°C for 30 s and 72°C for 45 s, followed by a final extension step of 72°C for 10 min; (2) *CTNNB1*: an initial denaturation step at 95°C for 5 min, 35 cycles of 95°C for 30 s, 49°C for 30 s and 72°C for 45 s, and a final extension step of 72°C for 10 min; (3)*WISP1*: an initial denaturation step at 95°C for 5 min; 35 cycles of 95°C for 30 s, 53°C for 30 s and 72°C for 45 s, and a final extension step of 72°C for 10 min. The thermal cycling was performed using the GeneAmp 9700 system (Applied Biosystems, Foster City, USA). PCR products were further digested by restriction enzymes according to the manufacturer's instructions. Sequences of the primer pairs, annealing temperatures, lengths of PCR products, and restriction enzymes for the three SNPs were shown in Table 4. After digestion, resulting fragments were separated on 3% agarose gels and stained with ethidium bromide for further analysis. A total of 10% of the genotyped samples were randomly duplicated and results were 100% concordant, and there were at least one positive and one negative control per 96-well DNA plate in our test. The PCR products were also sequenced by ABI 3730XL sequencer (Applied Biosystems, Foster City, USA) to detect the genotype (Figures 1, 2, 3).

**Table 4:** The primer sequences for each SNPs in Wnt signaling pathway

Polymorphism	PCR Primers (5′→3′)	Denaturation temperature	Products length	Restriction endonuclease
rs7832767	Forward: GAGTTCCACCCTCAATCTGT	51°C	676bp	*Tfil*
	Reverse: TTCCAGGGATGGTCTGTTAT			
rs2293303	Forward: TTGTTGACACCCTGACTCTT	49°C	349bp	*BsaBI*
	Reverse: TACAAATAGCCTAAACCACTC			
rs16893344	Forward: AGTCCCTGCCCGACAGAGTT	53°C	341bp	*Acel*
	Reverse: CTGATACAGGAGGGAGGATG			

SNP, single nucleotide polymorphism; PCR, polymerase chain reaction

### Statistical analysis

We analyzed whether genotype frequencies of all studied SNPs in control subjects were in Hardy-Weinberg equilibrium using the goodness-of-fit *χ*^2^ analysis. The differences in the continuous variables [shown as mean ± standard deviation (SD)] between the MI patients and the control subjects were assessed using an independent-sample t-test. Categorical variables and distribution of genotypes and alleles were shown as numbers and percentages (%), and the differences between the two groups were tested by χ2 test or Fisher's exact test. Moreover, logistic regression analysis was performed to assess the contribution of a certain genotype to MI. ORs and 95% CIs were calculated to determine the strength of the association between SNPs and MI. Multivariate analysis was conducted after adjustment for age, gender, smoking, drinking status, BMI, SBP, DBP, FPG, TG, TC, Uric acid, HDL-C, LDL-C, BUN, Cr. All statistical analyses were performed with using SPSS version 22.0 for Windows (SPSS Inc., USA), and statistical significance was established at an alpha level of 0.05.
